# Early prediction of outcome after severe traumatic brain injury: a simple and practical model

**DOI:** 10.1186/s12873-016-0098-x

**Published:** 2016-08-24

**Authors:** Sandro Rizoli, Ashley Petersen, Eileen Bulger, Raul Coimbra, Jeffrey D. Kerby, Joseph Minei, Laurie Morrison, Avery Nathens, Martin Schreiber, Airton Leonardo de Oliveira Manoel, the ROC Investigators

**Affiliations:** 1 Department of Trauma, St Michael’s Hospital, Toronto, ON Canada; 2 Department of Biostatistics, University of Washington, Seattle, WA USA; 3 Department of Surgery, Harborview Medical Center, University of Washington, Seattle, WA USA; 4 Department of Surgery, University of California San Diego, San Diego, CA USA; 5 Department of Surgery, University of Alabama at Birmingham, Birmingham, AL USA; 6 Department of Surgery, University of Texas Southwestern Medical Center, Dallas, TX USA; 7 Division of Emergency Medicine, University of Toronto, Toronto, ON Canada; 8 Division of General Surgery, Sunnybrook Health Sciences Centre, Toronto, ON Canada; 9 Department of Emergency Medicine, Oregon Health & Science University, Portland, OR USA; 10 Neuroscience Research Program, Keenan Research Center of the Li Ka Shing Knowledge Institute of St. Michael’s Hospital, 30 Bond Street, Toronto, ON M5B 1W8 Canada

**Keywords:** Traumatic brain injury, Recovery, Outcome measures, Prognostic models

## Abstract

**Background:**

Traumatic brain injury (TBI) is a heterogeneous syndrome with a broad range of outcome. We developed a simple model for long-term outcome prognostication after severe TBI.

**Methods:**

Secondary data analysis of a large multicenter randomized trial. Patients were grouped according to 6-month extended Glasgow outcome scale (eGOS): poor-outcome (eGOS ≤ 4; severe disability or death) and acceptable outcome (eGOS > 4; no or moderate disability). A prediction decision tree was built using binary recursive partitioning to predict poor or acceptable 6-month outcome. Comparison to two previously published and validated models was made.

**Results:**

The decision tree included the predictors of head Abbreviated Injury Scale (AIS) severity, the Marshall computed tomography score, and pupillary reactivity. All patients with a head AIS severity of 5 were predicted to have a poor outcome. In patients with head AIS severity < 5, the model predicted an acceptable outcome for (1) those with Marshall score of 1, and (2) those with Marshall score above 1 but with reactive pupils at admission. The decision tree had a sensitivity of 72.3 % (95 % CI: 66.4–77.6 %) and specificity of 62.5 % (95 % CI: 54.9–69.6 %). The proportion correctly classified for the comparison models was similar to our model. Our model was more apt at correctly classifying those with poor outcome but more likely to misclassify those with acceptable outcome than the comparison models.

**Conclusion:**

Predicting long-term outcome early after TBI remains challenging and inexact. This model could be useful for research and quality improvement studies to provide an early assessment of injury severity, but is not sufficiently accurate to guide decision-making in the clinical setting.

## Background

Traumatic brain injury (TBI) remains the leading cause of death and significant disability after severe blunt trauma [1]. It is estimated that 3.2 million people in the American continent live with disabilities caused by TBI [2]. Annually, the cost of TBI amounts to more than $35 billion in the United States alone [3–5].

In severe TBI, more than in any other injury, the preoccupation with long-term functional neurological outcome permeates many of the early decisions and interventions offered to these patients. Traumatic brain injury consists of a heterogeneous group of patients and injuries, in whom individual neurological recovery is difficult to predict at any time, but particularly early after admission. Accurate and useful prediction models to estimate neurological recovery is few and most only applicable when used days after the injury [6, 7]. The ability to reliably predict neurological recovery early could allow customization of medical decisions for physicians and families during initial resuscitation, from diagnosis to interventions. It could also reduce unwarranted decisions to withdraw life-supporting measures due to the perception of unfavorable neurological recovery [8, 9], help stratify patients into protocols and clinical trials, and assist with quality evaluation and improvement programs among many other utilities.

A variety of scales and models have been used to predict outcome after TBI, with many limitations besides not being applicable early after admission [6, 7]. Anatomic injury scales, such as Injury Severity Score (ISS) and the Abbreviated Injury Scale (AIS) are widely adopted predictors of trauma outcome. These scales are calculated by trained technicians and help convey the threat to life but fail to discriminate long-term neurological outcome. The simpler, easy to remember and vastly adopted Glasgow Coma Scale (GCS) [10] is also associated with mortality but not functional outcome. Other models have been criticized for being developed using small sample sizes, single center samples, lacking validation and for not being practical for use during early resuscitation [6, 7]. Some of these limitations were overcome by recent prognostic models developed through the IMPACT (International Mission for Prognosis and Analysis of Clinical Trials in TBI) [11] and CRASH (Corticosteroid Randomization After Significant Head Injury) [2] studies. These models were developed on large data sets and have good discriminatory power to predict neurological outcome at 6 months. The complexity of these models varies, and the calculations utilize clinical features, imaging and laboratorial measurements available soon after admission.

Using data from a recently completed, large, multicenter, double-blinded, randomized, placebo-controlled clinical trial [1], we developed a prognostic model for severe TBI using computed tomography (CT) scan and other readily available early parameters. To this end, we used recursive partitioning to build an easily interpretable model that predicts neurological outcome at 6 months using a limited number of patient characteristics available soon after injury.

## Methods

### Study design and setting

This study is a secondary analysis of data from a multicenter, double blind, randomized, placebo-controlled clinical trial conducted by the Resuscitation Outcomes Consortium between May 2006 and May 2009 (clinicaltrials.gov Identifier: NCT00316004). The trial involved 114 North American emergency medical services agencies and was conducted in two cohorts of patients: those with hypovolemic shock secondary to blunt or penetrating trauma and those with severe blunt TBI. This study is restricted to the second cohort of patients with severe blunt TBI. The objective of the trial in this cohort was to determine whether administering hypertonic fluids in the pre-hospital setting could improve long-term neurologic outcome as measured by 6-month eGOS. Additional details about the trial are available elsewhere [1].

### Population

The trial included patients aged 15 years or older with blunt trauma and severe TBI defined as a pre-hospital GCS of 8 or less who were determined to not be in hypovolemic shock. For this study, we excluded those patients who died within 24 h of emergency department (ED) admission, since this is a different population from long-term survivors, and the goal of this analysis was to predict outcome at 6-month. We also excluded patients with unknown survival status at 24 h, those without a blunt injury, and those who did not have a head CT.

### Outcome

The outcome of interest was the patient’s functional neurological status at 6 months post-injury, which we quantified using 6-month eGOS. Extended GOS was collected through a structured telephone survey of patients; a family member or caregiver was allowed to respond when the patient was unable to complete the survey. As in the original trial, we classified patients on the basis of whether they had a poor outcome (eGOS ≤ 4; severe disability or death) or an acceptable outcome (eGOS > 4; no or moderate disability). The primary analysis of this trial [1] used imputed 6-month eGOS as the outcome for patients missing 6-month eGOS. Similarly, those with missing 6-month eGOS in our study were assigned their average imputed eGOS from the primary analysis of the trial. The imputations were done using multiple hot deck imputations with 20 imputations [1].

### Predictors

In building our predictive model, we were interested in considering patient information collected upon arrival to ED including the results of the first head CT scan. The predictors we considered were pupil reactivity at ED admission (none, 1, or 2 reactive pupils), first systolic blood pressure (SBP) measurement in the ED, age, sex, Marshall score from the first head CT (measured on a scale from 1 to 6) [12], first GCS motor score in the ED, the head AIS severity (measured on a scale from 0 to 6), and the head AIS category. Head AIS scores were sorted into head AIS categories based on the region and type of injury. These categories include brainstem, cerebellar, contusion, diffuse, epidural, intraventricular hemorrhage (IVH), subarachnoid hemorrhage (SAH), and subdural hematoma (SDH). Each patient was classified as having or not having an injury for each of these categories. No requirement as to the severity of the injury was made as the minimum severity of a clinically important injury may vary between categories. As mentioned previously, this model was developed excluding patients who died within 24 h. Therefore, this rule would only be applicable ≥ 24 h.

### Statistical analysis

We used descriptive statistics to compare patient characteristics between those with a poor 6-month outcome (eGOS ≤ 4) and an acceptable 6-month outcome (eGOS > 4). Additionally, we considered how eGOS varied across observed combinations of Marshall score [12] and head AIS severity.

Our predictive model took the form of a decision tree [13], in which the patient population was repeatedly split on the basis of predictors into groups that were more and more homogeneous with respect to 6-month outcome (eGOS ≤ 4 or > 4). We used binary recursive partitioning to derive the decision tree(13). The tree classified patients into poor or acceptable 6-month outcome (eGOS ≤ 4 or > 4) based on patient characteristics collected soon after ED admission and the results of the first head CT scan. Recursive partitioning is useful when there are many potential complex interactions between the predictors of interest.

Observations were randomly split into training and validation sets, which contained 60 and 40 % of the observations respectively. The model was fit using the training set and the optimal size of the tree (i.e. number of splits) was determined using 10-fold cross-validation with the optimal tree of a given size being that which minimized the classification error, the proportion of patients whose predicted outcome status (eGOS ≤ 4 or > 4) was incorrect.

Two different techniques for handling missingness of the predictors were used: single imputation and surrogate splits. Single imputation was used, in lieu of multiple imputations, due to the difficulty of obtaining a single, simple decision rule when using multiple imputations. Alternatively, surrogate splits is a technique where a variable that is highly correlated with the splitting variable is used when the splitting variable is missing. Missingness of the outcome was handled through assigning patients their average imputed eGOS as described above.

The decision tree was evaluated in the separate validation set, which contained the remaining 40 % of patients in our data set. We calculated sensitivity, specificity, positive predictive value, negative predictive value, and percent correctly classified, as well as 95 % confidence intervals for each of these. These measures were compared to those obtained from using two previously validated models to predict 6-month outcome (eGOS ≤ 4 or > 4) after TBI, which were derived using data from 11 different studies and validated using data from the Medical Research Council CRASH Trial. We specifically compared to the *core* model, which incorporated age, GCS motor score, and pupil reactivity, and the *extended* model, which additionally incorporated hypotension status, hypoxia status, Marshall score, presence of traumatic subarachnoid hemorrhage, and presence of epidural hematoma. Both models provided predicted probabilities of eGOS ≤ 4. We dichotomized the predictions based on whether or not the predicted probabilities were above a certain threshold, which was chosen to be the threshold that maximized the percent correctly classified in the training set. Lastly, we compare the three models using receiver operating characteristic (ROC) curves, which illustrate the performance of the models for all possible thresholds for dichotomizing the predicted probabilities of the outcome. All statistical analyses were performed using R version 3.0.2 and the rpart library version 4.1–8.

## Results

### Descriptive statistics

**Fig. 1:**
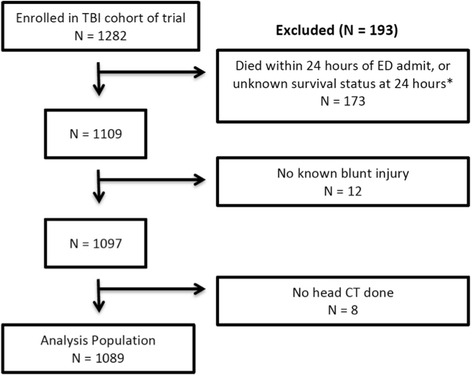
The refinement of the trial population to the analysis population. *Nine of these patients had unknown survival status as they refused consent

Of the 1282 patients enrolled in the TBI cohort of the trial, 1089 patients were included in our analysis with 193 (15 %) of those enrolled being excluded due to death within 24 h of ED admission, unknown 24-h survival status, no blunt injury, and no head CT (Fig. 1). Of those with non-missing eGOS, 456 (50 %) had a poor 6-month outcome (eGOS ≤ 4). There were 181 patients (17 %) with a missing eGOS – 90 % of these had an average imputed eGOS above 4. Thus including those with imputed eGOS, 43 % of patients in our analysis had a poor 6-month outcome.

Table 1 compares characteristics of predictors between those with poor versus acceptable 6-month eGOS (≤4 or > 4). The most striking differences between the two groups were related to higher head AIS and Marshall scores and absence of pupillary reactivity among those with a poor neurological outcome (eGOS ≤ 4).

**Table 1:** Summary of the predictors by 6-month eGOS^a^

	eGOS ≤ 4 (*n* = 474)	eGOS > 4 (*n* = 615)
Age (years)	42 (19)	34 (15)
Gender: Male	351 (74 %)	486 (79 %)
SBP, first ED measurement	141 (33)	142 (25)
GCS-motor, first ED measurement Missing	2.1 (1.7) 0 (0 %)	2.8 (2.1) 2 (0.3 %)
Categorized head AIS injuries		
Brainstem Cerebellar Contusion Diffuse Epidural IVH SAH SDH Other	38 (8.0 %) 16 (3.4 %) 156 (33 %) 115 (24 %) 30 (6.3 %) 86 (18 %) 168 (35 %) 184 (39 %) 14 (3.0 %)	6 (1.0 %) 14(2.3 %) 120 (20 %) 62 (10 %) 42 (6.8 %) 34 (5.5 %) 145 (24 %) 117 (19 %) 8 (1.3 %)
Head AIS severity		
No injury Minor Moderate Serious Severe Critical *Missing*	59 (12 %) 1 (0.2 %) 15 (3.2 %) 47 (9.9 %) 112 (24 %) 232 (49 %) 8 (1.7 %)	152 (25 %) 5 (0.8 %) 97 (16 %) 106 (17 %) 142 (23 %) 105 (17 %) 8 (1.3 %)
Marshall score, first head CT		
Diffuse Injury I (no visible pathology) Diffuse Injury II Diffuse Injury III (swelling) Diffuse Injury IV (shift) Evacuated mass lesion Non-evacuated mass lesion *Missing*	71 (15 %) 186 (39 %) 80 (17 %) 23 (4.9 %) 104 (22 %) 10 (2.1 %) 0 (0 %)	297 (48 %) 235 (38 %) 40 (6.5 %) 7 (1.1 %) 33 (5.4 %) 2 (0.3 %) 1 (0.2 %)
Pupil reactivity at ED admission		
0 pupils 1 pupil 2 pupils *Missing*	159 (34 %) 34 (7.2 %) 256 (54 %) 25 (5.3 %)	79 (13 %) 24 (3.9 %) 490 (80 %) 22 (3.6 %)

^a^Summaries include: mean (standard deviation) for continuous variables and n (%) for binary variables

Average imputed 6-month eGOS used for those missing eGOS

Table 2 compares the mean 6-month eGOS across the observed combinations of Marshall score and head AIS severity. There were no patients with a head AIS severity of 6 in our analysis population. Note that the imputed eGOS was used for those missing eGOS, however those with missing Marshall score or head AIS severity were excluded (*n* = 16). Those patients with a Marshall score of Diffuse Injury I tended to do well at 6 months (eGOS > 4), regardless of their head AIS severity. Aside from that, 6-month outcomes tended to worsen with higher head AIS severities and higher Marshall scores.

**Table 2:** Mean eGOS for different combinations of head AIS severity and Marshall score with number of patients per cell indicated in parentheses (NA indicated for cells without observations)

		Marshall score					
		Diffuse Injury I	Diffuse Injury II	Diffuse Injury III	Diffuse Injury IV	Evacuated mass lesion	Non-evacuated mass lesion
Head AIS severity	No injury (Score of 0)	6.1 (184)	4.1 (18)	1.7 (3)	NA	5.9 (2)	2.0 (4)
	Minor (Score of 1)	5.8 (6)	NA	NA	NA	NA	NA
	Moderate (Score of 2)	7.0 (106)	4.7 (3)	1.0 (1)	4.0 (1)	3.0 (1)	NA
	Serious (Score of 3)	6.6 (38)	5.6 (92)	5.5 (16)	NA	4.2(6)	8.0 (1)
	Severe (Score of 4)	5.2 (8)	5.5 (172)	4.3 (35)	4.4 (9)	3.3 (27)	1.0 (3)
	Critical (Score of 5)	5.0 (15)	4.1 (134)	3.2 (65)	2.6 (20)	2.7 (100)	1.0 (3)

The predictors of age, sex, first ED SBP, and head AIS categories do not have any missing data. The other predictors had limited amounts of missingness: first ED GCS motor (0.2 % missing), head AIS severity (1.5 %), Marshall score (0.1 %), and pupil reactivity (4.3 %). Overall, 94.2 % of patients had no missing data for any of the predictors.

### Decision tree selection

**Fig. 2:**
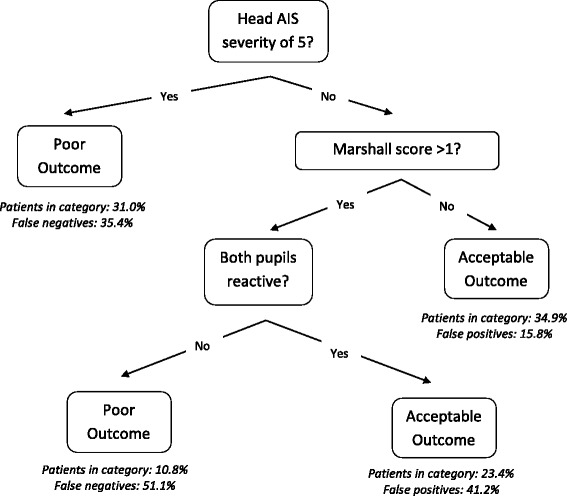
Decision tree for predicting poor (6-month eGOS ≤ 4) or acceptable (6- month eGOS > 4) neurological outcome for TBI patients. The percent of patients falling into each category, as well as the false positive or negative rate, is indicated for the validation data set. Note that our model applies only to those with a head AIS severity of 5 or lower, as our study population did not include any patients with a head AIS severity of 6

The final decision tree is included in Fig. 2. All patients with a head AIS severity of 5 were predicted to have a poor outcome (eGOS ≤ 4). Among those with a head AIS severity less than 5, the tree predicts an acceptable outcome (eGOS > 4) for two groups of patients: (1) those with a Marshall score of 1, and (2) those with a Marshall score above 1 but with reactive pupils at ED admission. Note that the trees selected were identical whether surrogate splits or single imputation was used to address the missingness of predictors.

### Decision tree validation

In Table 3, we present the classification performance of the proposed decision tree on the validation data set [13]. The results presented correspond to using the single imputation models from the training set to impute any missing predictors in the validation data set. However, the results from using surrogate splits to handle missingness were very similar. We also present the proportion of patients in each final category of the decision tree, as well as the percent of false positives or negatives, for the validation data set in Fig. 2. Additionally, we present the performance results in comparison to the previously validated models from in Table 3 [11]. The proportion correctly classified for these models is similar to our proposed model, while our model has higher specificity (our model vs. core: *p* = 0.007; extended: *p* = 0.001) and lower sensitivity (our model vs. core: *p* = 0.002; extended: *p* < 0.001) than the comparison models. This is consistent with our model being more apt at correctly classifying those with a poor outcome (eGOS ≤ 4), but more likely to misclassify those who in truth have an acceptable outcome (eGOS > 4).

**Table 3:** Summary accuracy measures in the validation sample for our proposed model and two of the models proposed in [2]^a^

	Our proposed model Estimate (95 % confidence interval)	Previously validated core model Estimate (95 % confidence interval)	Previously validated extended model Estimate (95 % confidence interval)
Sensitivity	72.3 % (66.4–77.6 %)	83.8 % (78.7–88.0 %)	92.7 % (88.6–95.4 %)
Specificity	62.5 % (54.9–69.6 %)	47.7 % (40.2–55.4 %)	44.3 % (36.9–52.0 %)
Positive predictive value	74.0 % (68.1–79.2 %)	70.3 % (64.8–75.3 %)	71.1 % (65.9–75.8 %)
Negative predictive value	60.4 % (52.9–67.5 %)	66.7 % (57.6–74.7 %)	80.4 % (70.9–87.5 %)
Correct classification	68.3 % (63.7–72.6 %)	69.3 % (64.7–73.5 %)	73.2 % (68.7–77.2 %)

^a^In calculating these measures, we used ‘positive’ to denote an acceptable outcome (eGOS > 4)

**Fig. 3:**
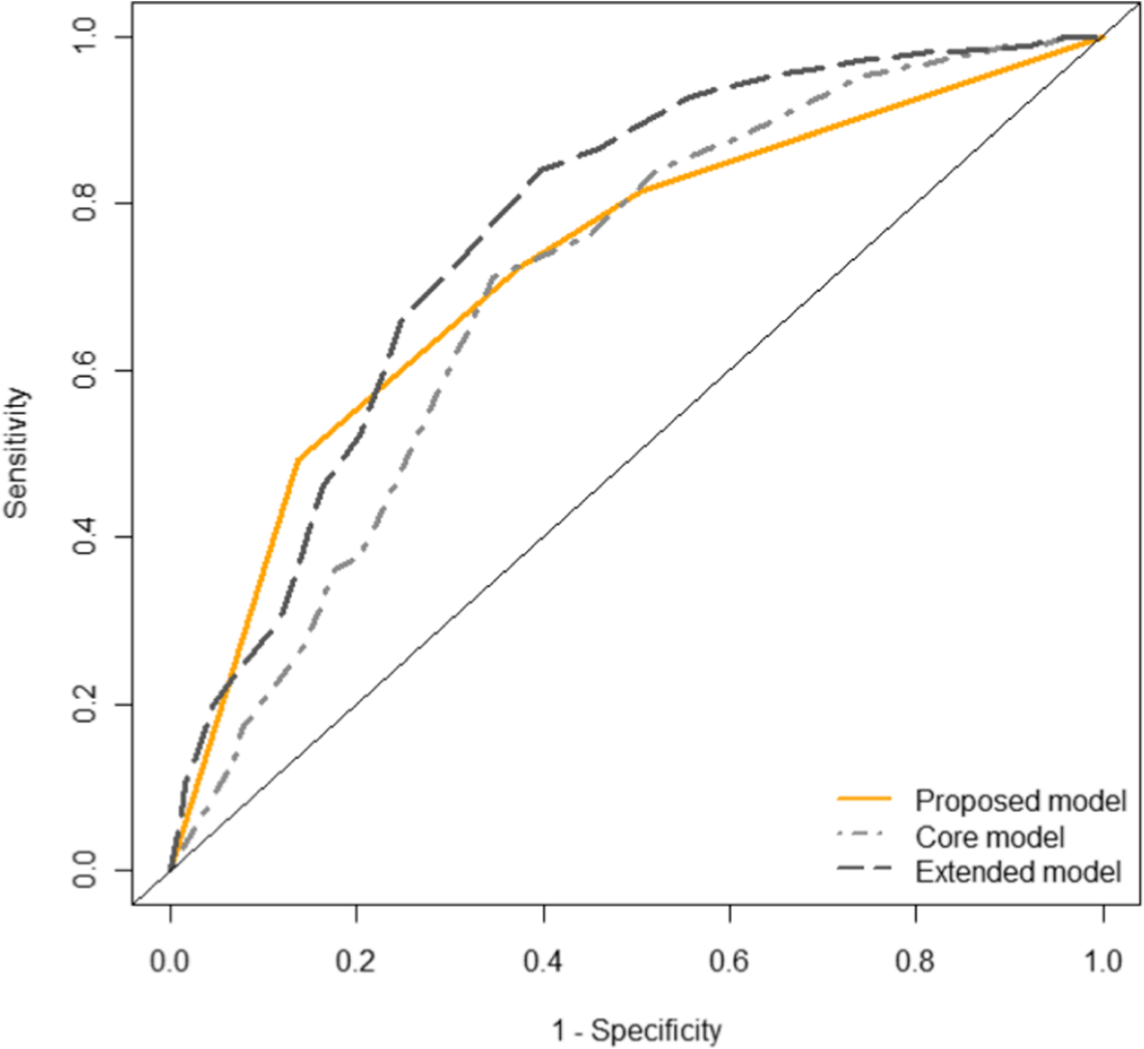
ROC curves comparing the performance in the validation set of the proposed model with two previously validated models. Sensitivity is the proportion of those with an acceptable outcome who were correctly predicted to have an acceptable outcome. Specificity is the proportion of those with a poor outcome who were correctly predicted to have a poor outcome

While the results for Table 3 correspond to dichotomizing predicted probabilities based on a specific threshold, we can also consider the performance for any threshold and plot the corresponding sensitivity and specificity in a ROC curve. The ROC curves for the three models are shown in Fig. 3. The proposed model performed the best in comparison to the two other models when thresholds for dichotomization were chosen to favor high specificity over high sensitivity. The area under the curve (AUC) for our proposed model was 0.67 (95 % CI: 0.63–0.72). The AUCs for the comparison models of core and extended were 0.66 (95 % CI: 0.61–0.70) and 0.69 (95 % CI: 0.66–0.73), respectively. Comparing our model to the previously published models, our model had an AUC that was 0.016 higher (95 % CI:−0.034–0.066; *p* = 0.52) than the core model and 0.011 lower (95 % CI:−0.034—0.056; *p* = 0.63) than the extended model.

## Discussion

Early management of patients with severe TBI is among the most challenging issues in trauma. Traumatic brain injury is the leading cause of death, but more relevant to this work is that up to 50 % of those with severe TBI will have long-term sequelae and up to 30 % develop devastating long-term neurological deficits. Furthermore, it is notoriously difficult to identify patients that will have a poor neurological recovery during the early phases of resuscitation [14–18]. We found that a decision tree approach could predict with acceptable certainty, which patients with severe blunt TBI will have a poor or acceptable 6-month neurological outcome.

The model was developed using a population of 1089 patients with severe TBI enrolled in a recently completed randomized controlled trial. Forty-three percent of the patients had a poor 6-month outcome (eGOS ≤ 4). A decision tree built using binary recursive partitioning [13] was used considering its usefulness in situations where many potential complex interactions occur, as expected in this population. The final decision tree included the three predictors of head AIS severity, Marshall score, and pupil reactivity, which were selected as a part of the recursive partitioning process from eight different potential predictors available shortly after ED arrival.

The decision tree predictive model we developed is simple, easy-to-remember, constructed on the first head CT scan and admission characteristics, and is capable of predicting the likelihood of unfavorable outcome at 6 months. It starts by determining whether the patient with severe TBI has an AIS head score of 5. The head AIS severity of 5 is bestowed to patients alive with large (>1 cm thick) subdural or epidural hematomas or massive/extensive intra cerebral hemorrhage or contusions. The presence of a head AIS severity of 5 is associated with poor 6-month functional neurological outcome (eGOS ≤ 4). For the patients with head AIS severity less than 5, we split patients into those with a Marshall score of 1 and those with a score greater than 1. The Marshall score of 1 is defined as a head CT scan with no visible pathology, and these patients are predicted to have an acceptable 6-month outcome. For those with any abnormality on the head CT scan (Marshall score >1), the next and final predictive factor is the presence of bilateral reactive pupils, which is associated with acceptable outcome versus poor outcome if one or both pupils are not reactive.

The three prognostic indicators: anatomical head injuries scored by the AIS, head CT scan and pupil reactivity [16] have been used in other predictive models. The final head AIS severity is rarely estimated in the first hours following hospital admission, as well as the definitive radiology interpretation of the head CT scan, that in many trauma centers is only available the following day. Nevertheless, the presence of extensive anatomical injuries to the head (AIS severity of 5), the identification of head CT scan as having no visible pathology (Marshall score of 1) and pupil reaction are consistently sought and recognized by clinicians during resuscitation, and used to make medical decisions. Thus the expectation is that clinicians with some experience in trauma resuscitation would have no difficulties in using this predictive model in practice.

The predictive model has a good discrimination between patients with poor and acceptable outcome. The internal validation was performed in a separate set of patients containing 40 % of all patients from our data set. The percent correctly classified (Table 3) was similar to two predictive models described by Steyerberg et al. [11] Relative to the comparison models, our model had a higher specificity, i.e. was more apt at correctly classifying those with poor outcome, but a lower sensitivity, i.e. more likely to misclassify those with acceptable outcome. The choice to compare our model to these two other predictive models comes from the fact that those models are similar to ours in aiming to prognosticate 6-month outcomes after severe TBI using readily available indicators that have been validated. Our model differs from theirs in that it is not designed to predict mortality, consists of a simple decision tree while theirs are developed using logistic regression and our predictions are readily available without requiring calculations or some form of computer support.

Any predictive model must be used with caution, in particular decision-making for individual patients. A recent Canadian multicenter cohort study [9] demonstrated that most deaths after severe TBI (45 to 86.8 %) were due to withdrawal of life-supporting measures, often based on perception of unfavorable chances of meaningful neurological recovery. A significant proportion of the deaths due to withdrawal occurred early, within 3 days of injury, and varied significantly by center (odds ratios varied between 0.42 and 2.4, *p* = 0.0001). The expressed caution from this study was that young and otherwise healthy TBI patients might have their life supporting measures needlessly withdrawn due to the perception of unfavorable neurological outcomes. In relation to our findings, our model had a lower sensitivity than the two previously validated models. That is, a smaller portion of those with acceptable 6-month outcomes were identified correctly using our model. This could have detrimental effects in the clinical setting if our model was used as justification for withdrawal of life support or further interventions. Thus we caution against using this model to guide decision-making in the clinical setting, as it is not yet accurate enough.

The present study has several limitations. The original study was designed to study the effect of pre-hospital administration of hypertonic fluids on the long-term (6-month) neurological outcome of patients with severe TBI without hypovolemic shock. While it should be noted, it is improbable that the administration of hypertonic fluids affected the neurological outcome and the development of the predictive model. Few variables and values were missing, as detailed in the results. Two different techniques for handling missingness were used, single imputation and surrogate splits. While these techniques are warranted, missingness remains another limitation.

## Conclusion

Predicting long-term neurological recovery early after head injury remains a major challenge. Using data from a large multicenter randomized controlled trial, we analyzed 1089 adult patients with severe TBI, no evidence of hemorrhagic shock and with at least a head CT scan performed. We then developed a prognostic decision tree capable of discriminating patients with poor or acceptable 6-month functional neurological outcome (eGOS ≤4 or >4). The decision tree prognostic model uses 3 indicators commonly sought and used by practicing clinicians during early resuscitation of patients with severe TBI: the extent of anatomical damage to the head (head AIS severity), the initial head CT scan (Marshall score) and pupil reactivity. This early predictive model could be useful for research and quality improvement, but caution should be exercised when using it for clinical decision-making.

## Abbreviations

TBI: Traumatic brain injury
eGOS: Glasgow outcome scale
AIS: Abbreviated Injury Scale
ISS: Injury Severity Score
GCS: Glasgow Coma Scale
IMPACT: International Mission for Prognosis and Analysis of Clinical Trials in TBI
CRASH: Corticosteroid Randomization After Significant Head Injury
CT: Computed tomography
ROC: Receiver operating characteristic
ED: Emergency department
SBP: Systolic blood pressure
IVH: Intraventricular hemorrhage
SAH: Subarachnoid hemorrhage
SDH: Subdural hematoma
AUC: Area under the curve

## References

[1] Bulger EM, May S, Brasel KJ, Schreiber M, Kerby JD, Tisherman SA, Newgard C, Slutsky A, Coimbra R, Emerson S, Minei JP, Bardarson B, Kudenchuk P, Baker A, Christenson J, Idris A, Davis D, Fabian TC, Aufderheide TP, Callaway C, Williams C, Banek J, Vaillancourt C, van Heest R, Sopko G, Hata JS, Hoyt DB, ROC Investigators (2010). Out-of-hospital hypertonic resuscitation following severe traumatic brain injury: a randomized controlled trial. JAMA.

[2] Perel P, Arango M, Clayton T, Edwards P, Komolafe E, Poccock S, Roberts I, Shakur H, Steyerberg E, Yutthakasemsunt S, MRC CRASH Trial Collaborators (2008). Predicting outcome after traumatic brain injury: practical prognostic models based on large cohort of international patients. BMJ.

[3] Humphreys I, Wood RL, Phillips CJ, Macey S (2013). The costs of traumatic brain injury: a literature review. Clinicoecon Outcomes Res.

[4] McGarry LJ, Thompson D, Millham FH, Cowell L, Snyder PJ, Lenderking WR, Weinstein MC (2002). Outcomes and costs of acute treatment of traumatic brain injury. J Trauma.

[5] Thompson K, Antony A, Holtzman A (2001). The costs of traumatic brain injury. NC Med J.

[6] Mushkudiani NA, Hukkelhoven CW, Hernández AV, Murray GD, Choi SC, Maas AI, Steyerberg EW (2008). A systematic review finds methodological improvements necessary for prognostic models in determining traumatic brain injury outcomes. J Clin Epidemiol.

[7] Perel P, Edwards P, Wentz R, Roberts I (2006). Systematic review of prognostic models in traumatic brain injury. BMC Med Inform Decis Mak.

[8] Turgeon AF, Lauzier F, Burns KE, Meade MO, Scales DC, Zarychanski R, Moore L, Zygun DA, McIntyre LA, Kanji S, Hébert PC, Murat V, Pagliarello G, Fergusson DA, Canadian Critical Care Trials Group (2013). Determination of neurologic prognosis and clinical decision making in adult patients with severe traumatic brain injury: a survey of Canadian intensivists, neurosurgeons, and neurologists. Crit Care Med.

[9] Turgeon AF, Lauzier F, Simard JF, Scales DC, Burns KE, Moore L, Zygun DA, Bernard F, Meade MO, Dung TC, Ratnapalan M, Todd S, Harlock J, Fergusson DA, Canadian Critical Care Trials Group (2011). Mortality associated with withdrawal of life-sustaining therapy for patients with severe traumatic brain injury: a Canadian multicentre cohort study. CMAJ.

[10] Teasdale G, Jennett B (1974). Assessment of coma and impaired consciousness. A practical scale. Lancet.

[11] Steyerberg EW, Mushkudiani N, Perel P, Butcher I, Lu J, McHugh GS, Murray GD, Marmarou A, Roberts I, Habbema JD, Maas AI (2008). Predicting outcome after traumatic brain injury: development and international validation of prognostic scores based on admission characteristics. PLoS Med.

[12] Marshall LF, Marshall SB, Klauber MR, Van Berkum Clark M, Eisenberg H, Jane JA, Luerssen TG, Marmarou A, Foulkes MA (1992). The diagnosis of head injury requires a classification based on computed axial tomography. J Neurotrauma.

[13] Breiman L, Friedman JH, Stone CJ, Olshen RA, Stone CJ. Classification and regression trees, Wadsworth, Belmont, CA, 1984. In: Chapman & Hall. New York: 1984. p. I41–I63.

[14] Roozenbeek B, Lingsma HF, Lecky FE, Lu J, Weir J, Butcher I, McHugh GS, Murray GD, Perel P, Maas AI, Steyerberg EW, International Mission on PrognosisAnalysis of Clinical Trials in Traumatic Brain Injury (IMPACT) Study Group, Corticosteroid Randomisation After Significant Head Injury (CRASH) Trial Collaborators, Trauma Audit and Research Network (TARN) (2012). Prediction of outcome after moderate and severe traumatic brain injury: external validation of the International Mission on Prognosis and Analysis of Clinical Trials (IMPACT) and Corticoid Randomisation After Significant Head injury (CRASH) prognostic models. Crit Care Med.

[15] McHugh GS, Engel DC, Butcher I, Steyerberg EW, Lu J, Mushkudiani N, Hernández AV, Marmarou A, Maas AI, Murray GD (2007). Prognostic value of secondary insults in traumatic brain injury: results from the IMPACT study. J Neurotrauma.

[16] Marmarou A, Lu J, Butcher I, McHugh GS, Murray GD, Steyerberg EW, Mushkudiani NA, Choi S, Maas AI (2007). Prognostic value of the glasgow coma scale and pupil reactivity in traumatic brain injury assessed pre-hospital and on enrollment: an IMPACT analysis. J Neurotrauma.

[17] Butcher I, Maas AI, Lu J, Marmarou A, Murray GD, Mushkudiani NA, McHugh GS, Steyerberg EW (2007). Prognostic value of admission blood pressure in traumatic brain injury: results from the IMPACT study. J Neurotrauma.

[18] Wong GK, Teoh J, Yeung J, Chan E, Siu E, Woo P, Rainer T, Poon WS (2013). Outcomes of traumatic brain injury in Hong Kong: validation with the TRISS, CRASH, and IMPACT models. J Clin Neurosci.

